# Detection of Diurnal Variation of Tomato Transcriptome through the Molecular Timetable Method in a Sunlight-Type Plant Factory

**DOI:** 10.3389/fpls.2016.00087

**Published:** 2016-02-08

**Authors:** Takanobu Higashi, Yusuke Tanigaki, Kotaro Takayama, Atsushi J. Nagano, Mie N. Honjo, Hirokazu Fukuda

**Affiliations:** ^1^Graduate School of Life and Environmental Sciences, Osaka Prefecture UniversitySakai, Japan; ^2^Graduate School of Engineering, Osaka Prefecture UniversitySakai, Japan; ^3^Faculty of Agriculture, Ehime UniversityMatsuyama, Japan; ^4^Faculty of Agriculture, Ryukoku UniversityOtsu, Japan; ^5^Center for Ecological Research, Kyoto UniversityOtsu, Japan; ^6^Precursory Research for Embryonic Science and Technology, Japan Science and Technology AgencyKawaguchi, Japan

**Keywords:** circadian clocks, molecular timetable method, stress-responsive genes, plant factory, tomato, transcriptome

## Abstract

The timing of measurement during plant growth is important because many genes are expressed periodically and orchestrate physiological events. Their periodicity is generated by environmental fluctuations as external factors and the circadian clock as the internal factor. The circadian clock orchestrates physiological events such as photosynthesis or flowering and it enables enhanced growth and herbivory resistance. These characteristics have possible applications for agriculture. In this study, we demonstrated the diurnal variation of the transcriptome in tomato (*Solanum lycopersicum*) leaves through molecular timetable method in a sunlight-type plant factory. Molecular timetable methods have been developed to detect periodic genes and estimate individual internal body time from these expression profiles in mammals. We sampled tomato leaves every 2 h for 2 days and acquired time-course transcriptome data by RNA-Seq. Many genes were expressed periodically and these expressions were stable across the 1st and 2nd days of measurement. We selected 143 time-indicating genes whose expression indicated periodically, and estimated internal time in the plant from these expression profiles. The estimated internal time was generally the same as the external environment time; however, there was a difference of more than 1 h between the two for some sampling points. Furthermore, the stress-responsive genes also showed weakly periodic expression, implying that they were usually expressed periodically, regulated by light–dark cycles as an external factor or the circadian clock as the internal factor, and could be particularly expressed when the plant experiences some specific stress under agricultural situations. This study suggests that circadian clock mediate the optimization for fluctuating environments in the field and it has possibilities to enhance resistibility to stress and floral induction by controlling circadian clock through light supplement and temperature control.

## Introduction

Recently agricultural technologies have been rapidly developing, for example, application of information and communication technology, automation, and cultivation in closed systems with controlled temperature, humidity, and light conditions. In particular, plant factories are the more recent and highly regarded. These are of two types, closed-type, and sunlight-type. Both types have abilities of mass production, stable supply, and safe products. Furthermore, both types can control the cultivation environment artificially compared with open culture. Accordingly, there has been increased expectation of the ability to create cultivation control technologies for several cultivars according to various internal factors, which could be a key for high productivity or high quality. There is a real need for an exhaustive understanding of gene expression or metabolism to determine what these internal factors are, for example stress responses (Mittler and Blumwald, 2010). With this background, there have been great advances in technologies to analyze the genome, transcriptome, proteome, and metabolome. These analyses are increasingly user-friendly and their use in studies has increased rapidly. These molecular biological approaches have potential to solve several agricultural problems. In the case of fruit vegetables, there is a need to clarify some of the internal factors responsible for such physiological disorders as fruit cracking, blossom-end rot (Cuartero and Fernández-Muñoz, 1999; Guichard et al., 2001), and floral induction (Lifschitz et al., 2006; Corbesier et al., 2007). These are also critical issues for yield improvements.

Searching of these internal factors through biological statistical analysis has shown the usefulness of biological models (Takahashi et al., 2012; Guanter et al., 2014). However, because many genes are expressed periodically, diurnal variation must be considered when choosing a time for measurement (Harmer et al., 2000). These periodic expressions are generated by the day–night environment as the external factor and the circadian clock as the internal factor. Most living organisms have a circadian clock system and that of plants orchestrates physiological events such as gene expression, protein phosphorylation, chloroplast movement, stomatal opening, and flowering (Barak et al., 2000). The circadian clock system has three components (Farré and Weise, 2012): input pathways receive the external stimuli such as light and temperature fluctuations; the oscillator generates the endogenous circadian rhythms; and output pathways regulate general metabolism (e.g., of nitrogen and sugars). Endogenous circadian rhythms are generated by a number of genes; for example, *CCA1* (*CIRCADIAN CLOCK ASSOCIATED 1*), *TOC1* (*TIMING OF CAB EXPRESSION 1*), *LHY* (*LATE ELONGATED HYPOCOTYL*), and *PRRs* (*PSEUDO-RESPONSE REGULATORs*), which create feedback loops in each cell (Alabadí et al., 2001; Nakamichi et al., 2010, 2012; Haydon et al., 2011). These genes—including *PHYs*, which are the far-red light receptors; and *CRYs*, which are the blue light receptors (Pruneda-Paz and Kay, 2010)—receive signals from the input pathway and are expressed periodically with control of their own amplitude, period, and phase. It is well known that controlling the circadian rhythm enhances growth and herbivory resistance (Dodd et al., 2005; Goodspeed et al., 2012, 2013; Higashi et al., 2015), and the circadian clock can be controlled by external stimuli that change the light–dark cycle and by temperature fluctuation, because the circadian clock has characteristics of entrainment to the environment (Rensing and Ruoff, 2002; James et al., 2012; Fukuda et al., 2013). The circadian clock regulates hundreds of genes in Arabidopsis (*Arabidopsis thaliana*). Photosynthesis or phenylpropanoid biosynthesis genes are expressed periodically, with these genes peaking at different times such as subjective day and dawn under constant light (Harmer et al., 2000). These studies have shown that many genes generate circadian fluctuations without external stimuli. One recent study indicated that the day–night environment as the external oscillator and the circadian clock as the internal molecular oscillator activated transcription factors and mediated the circadian outputs (Haydon et al., 2011). The output adapts the circadian gene expression and entrains it to the external environment. This study suggests that the day–night environment as the external oscillator is a fast pathway and that the circadian clock as the internal molecular oscillator is a slow pathway for activation of transcriptome factors. Furthermore, hundreds of genes oscillate with circadian and circannual rhythms in rice (*Oryza sativa*) under field conditions (Nagano et al., 2012). They can generate comparatively stable rhythms under greatly variable conditions through the internal molecular oscillator. This indicates that in many cases we should consider the plant's internal rhythm and take care choosing a measurement time. Thus, very accurate analysis of diurnal variation will have application in development of biological models for yield improvements.

In this study, we tried to develop a highly accurate analysis of diurnal variation in tomato leaves in a sunlight-type plant factory as a basis for exhaustive study of the transcriptome and metabolome. We obtained time-course transcriptome data and used the molecular timetable method (Ueda et al., 2004) to analyze diurnal variation. Using these methods, we selected some periodic genes and estimated the phase of the circadian clock in tomato (internal time) from these periodic gene expression profiles.

## Materials and methods

### Plant materials and growing systems

Experiments were carried out using tomato (*Solanum lycopersicum* cv. Taiankichijitsu, Nanto Seed Co. Ltd., Nara, Japan) cultivated in a sunlight-type plant factory (4480 cm [W] × 2300 cm [D] × 500 cm [H]) in the Faculty of Agriculture, Ehime University, Japan. Individual plants are usually cultivated for a year; in this experiment, tomato seedlings were grown by Berg Earth Co. Ltd. (Ehime, Japan) and transplanted into rockwool cubes (10 cm [W] × 10 cm [D] × 6.5 cm [H], Grodan Delta, GRODAN, Roermond, The Netherlands) in August 2013. Rockwool cubes were placed on rockwool slabs (100 cm [W] × 20 cm [D] × 7.5 cm [H], Grotop Expert, GRODAN) at four per slab. The four rockwool cubes were placed at 25 cm intervals and watered using nutrient solution (Sonneveld, 1985). There were 20 slabs set in a line, with 28 lines per greenhouse. We sampled their leaves in January 2014. The light condition, relative humidity, and carbon dioxide concentration were ambient. Air temperature was maintained at 14°C during 18:00–8:00.

We sampled the fifth leaves every 2 h for 2 days, starting at 14:00 on 6 January 2014 and ending at 14:00 on 8 January 2014. We sliced leaf segments and stored them at 0°C with RNA-later solution (Qiagen, Valencia, CA, USA), an aqueous nontoxic tissue storage reagent that rapidly permeates tissue to stabilize and protect the integrity of RNA.

### RNA-seq assay and data analysis

We isolated total RNA using an RNeasy Plant Mini Kit (Qiagen). RNA quality was checked using an Agilent 2100 Bioanalyzer (Agilent Technologies, Palo Alto, CA, USA) and RNA quantity control was performed using a Qubit® 2.0 Fluorometer (Life Technologies, Carlsbad, CA, USA). We prepared a RNA-Seq library (Wang et al., 2011; Nagano et al., 2015) and sequencing was performed by BGI (Yantian District, Shenzhen, China). Then we obtained the sequence read files using a HiSeq 2000 sequencer (single end, 50 bp; Illumina, San Diego, CA, USA). These sequence data are available in the DDBJ Sequenced Read Archive (http://trace.ddbj.nig.ac.jp/DRASearch) under the accession number DRA003529 and DRA003530.

All reads of each sample were quality checked by FastQC and mapped using RSEM (RNA-Seq by Expectation Maximization) (Li and Dewey, 2011) with Bowtie2 software (Langmead and Salzberg, 2012) to the reference sequence. RSEM is a user-friendly software package able to accurately quantify transcript abundances. By these processes, we obtained the data of gene expression levels for each sample. Finally, we used the reads per kilobase per million mapped reads measure to normalize the gene expression for total read length and the number of sequencing reads. In this study, we calculated the average expression level of each sampling time for each genes. Then we cut off some genes whose average expression level is less than 5% of average expression level of whole genes and we identified 18,332 genes were significantly expressed.

### Molecular timetable method

Molecular timetable methods have been developed for administration of medicine to animals, with the objective to detect internal body time from periodic gene expression profiles (Ueda et al., 2004). As an outline of this methods, they selected “time-indicating genes,” which expressed periodically and represented internal body time from every 2 h for 2 days microarray data in the mouse liver. Then they developed gene expression profiles from time-indicating genes and estimated internal body time at each sampling time. To verify the performance of this methods, they calculated the sensitivity and specificity in the presence of different measurement noise and different number of time-indicating genes. We adapted this methods to use RNA-Seq data processing with cultivated plants.

First, we selected genes whose expression indicated periodicity and high amplitude from the time-course transcriptome data—these were time-indicating genes. To analyze periodicity, we prepared 1440 test cosine curves. These curves had different peaks (0–24 h) measured at increments of 1 min. We fitted test cosine curves to data from each time-course gene expression generated via RNA-Seq and calculated the correlation value (*r*) to identify the best-fitting cosine curve. The peak time of the best-fitting curve was estimated as the peak time for each gene. This estimated peak time defined as the molecular peak time. Thus, molecular peak time was estimated from a single gene, and all genes were estimated it individually. Then, to analyze amplitude, we calculated the average and standard deviation for every gene expression level. The amplitude value (*a*) was calculated as the standard deviation divided by the average of gene expression level.

Second, we plotted expression profiles of time-indicating genes. The number of time-indicating genes was determined by the cut-off values of *r* and *a*. We normalized the expression level of each time-indicating gene using its average and standard deviation. Normalized expression level was defined as the value of expression level minus average expression level, divided by the standard deviation. We then plotted expression profiles composed of the molecular peak time and normalized expression level for every sampling time. The horizontal axis indicated the molecular peak time and the vertical axis indicated normalized expression level.

Finally, the internal time was estimated by the plotted expression profile. We prepared 1440 test cosine curves with 1-min differences from each other and fitted them to the expression profiles. We identified the best-fitting cosine curve, and its peak time indicated the estimated internal time. Thus, the internal time was estimated from a number of periodic genes.

In this study, we selected 143 time-indicating genes by setting the cut-off values of *r* = 0.915 and *a* = 0.15 in whole genes and 150 time-indicating genes by setting the cut-off values of *r* = 0.635 and *a* = 0.15 in stress-responsive genes. In the previous study, they indicated that 150 time-indicating genes with 100% measurement noise could estimate internal time with high accuracy. Table S1 shows the measurement noise with 143 time-indicating genes in whole genes and 150 time-indicating genes in stress-responsive genes for each sampling time (see Supplementary Material). We calculated the standard deviation of the difference between a real and an estimated expression of all time-indicating genes as the measurement noise. Each measurement noise represent less than 100%. Thus, 143 and 150 time-indicating genes are enough to estimate the internal time with high accuracy.

### Mapman analysis

We used MapMan to categorize stress-responsive genes in tomato (Thimm et al., 2004). MapMan BINs were generated by Mercator (http://mapman.gabipd.org/web/guest/app/mercator) based on TAIR10 in Arabidopsis. MapMan software can be downloaded from http://mapman.gabipd.org/web/guest/mapman.

## Results

### Environmental conditions in a sunlight-type plant factory

**Figure 1:**
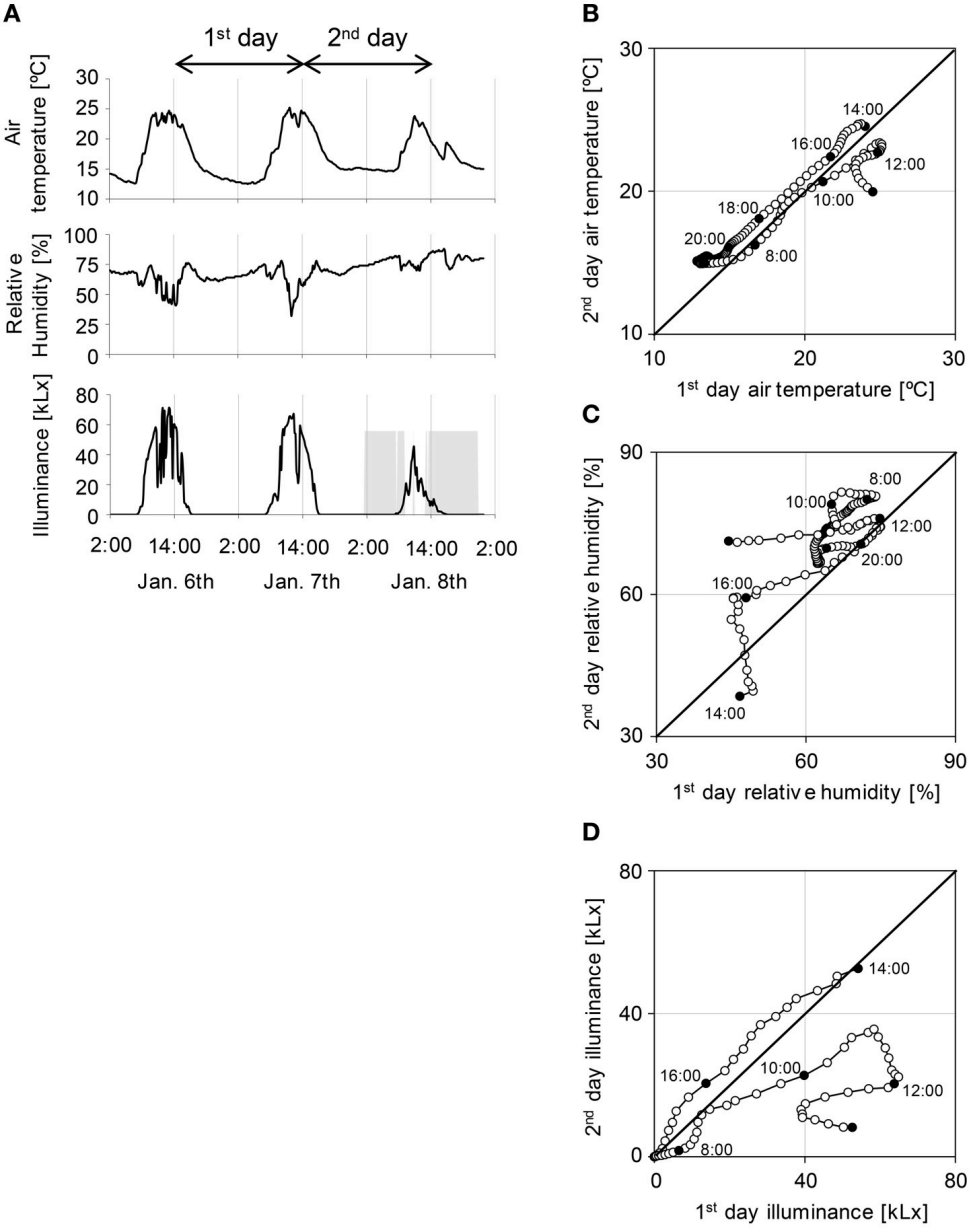
**Environmental conditions in a sunlight-type plant factory in Ehime University (A) and comparison of the 1st with the 2nd day for each environmental condition (B–D). (A)** Air temperature, relative humidity, and illuminance in a sunlight-type plant factory. Gray area of illuminance indicates precipitation. **(B–D)** Open circles indicate each values for every 10 min and filled circles indicate the sampling point. Sampling started at 14:00.

We sampled tomato leaves every 2 h during 6–8 January 2014 in a sunlight-type plant factory in the Faculty of Agriculture, Ehime University, Japan (Takayama et al., 2012). Because it was winter during the experiments, the air temperature, relative humidity, and illuminance were comparatively low and day–night cycle had short days (day of 10 h and night of 14 h) (Figure 1A). As it rained on 8 January, this caused decreases in air temperature and illuminance, and increased relative humidity. Air temperature was relatively stable over the 1st and 2nd days of the experiment (Figure 1B); in contrast, relative humidity, and illuminance were relatively unstable on each day (Figures 1C,D). These results show that relative humidity and illuminance were strongly influenced by the rainy day.

### Selection of time-indicating genes and development of these expression profiles

**Figure 2:**
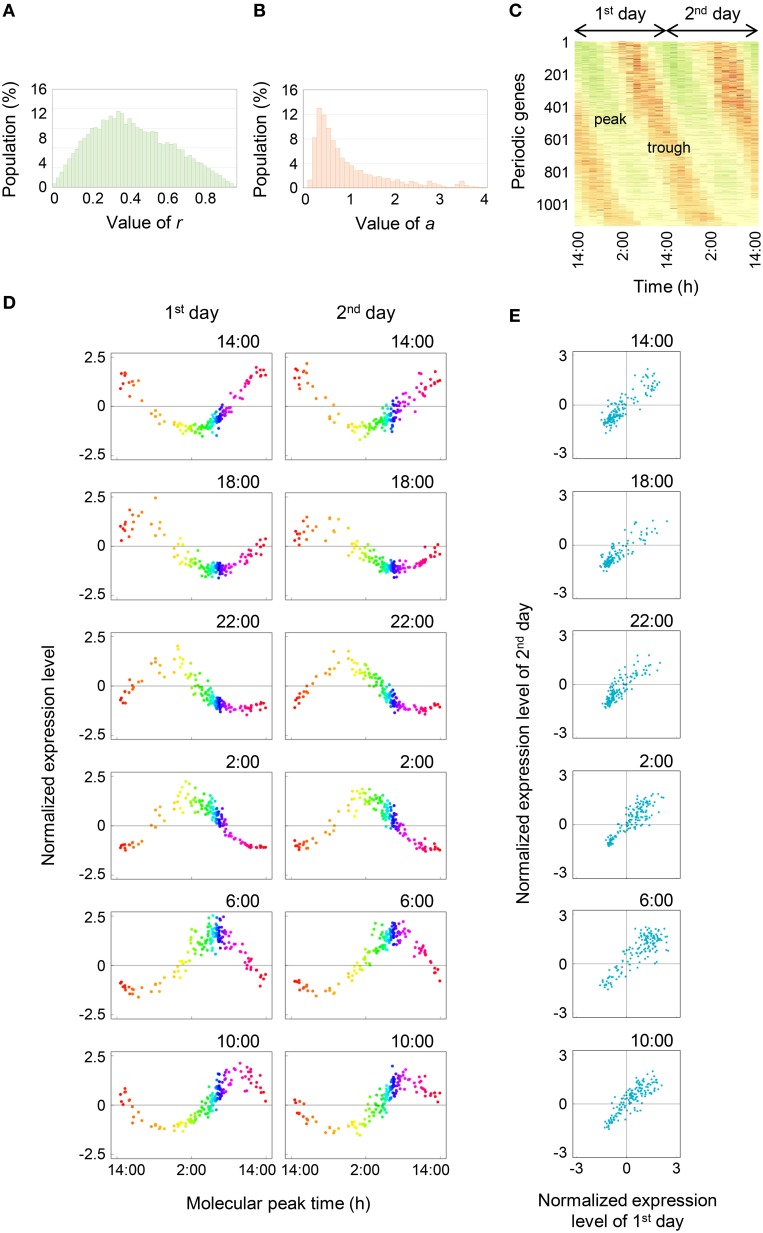
**Comprehensive analysis of diurnal oscillation in gene expressions. (A,B)** Histograms of the *r* and *a* values, respectively. **(C)** Oscillation behavior of time-indicating genes. Green area indicates the peak and red area indicates the trough. **(D)** Expression profiles of 143 time-indicating genes for the 1st and 2nd days. The time at the top right of the plot area indicates the sampling time. The range from orange to purple represents night-time and other colored areas represent day-time. **(E)** Correlations between normalized expression levels for the 1st and 2nd days. The time at the top right of the plot area indicates the sampling time.

**Figure 3:**
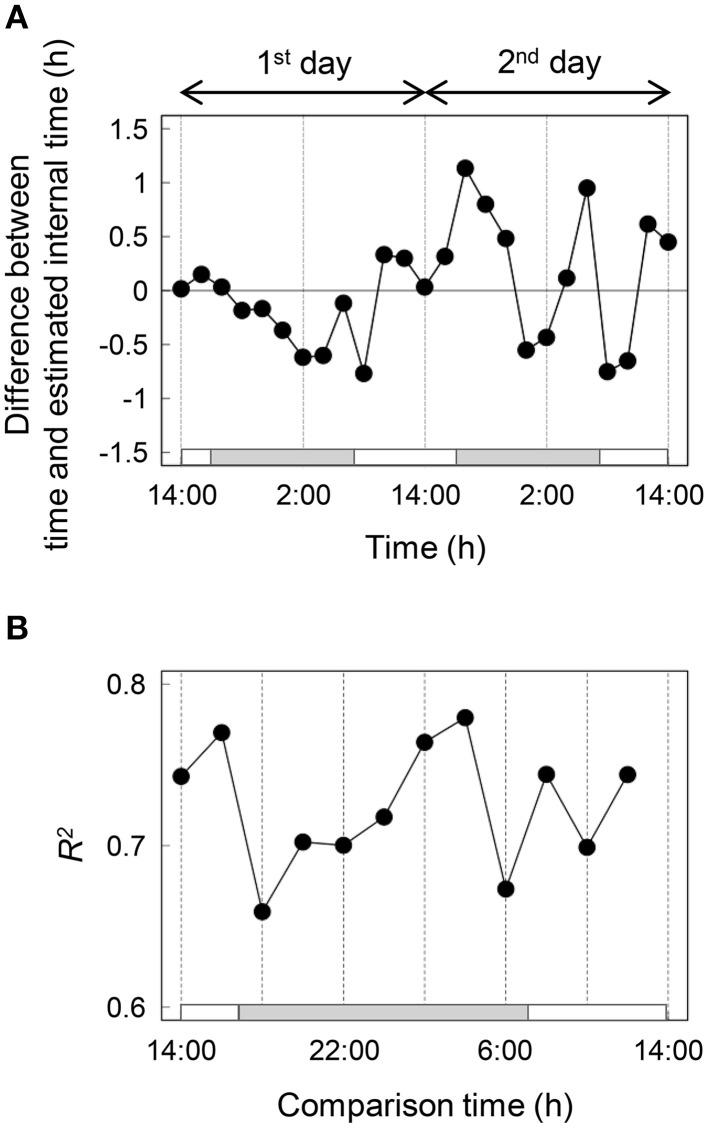
**Validation of the internal time estimation (A) and transition of the determination coefficient between normalized expression levels for the 1st and 2nd days (B)**. White and gray bars represent light and dark, respectively.

Then we analyzed the diurnal oscillation in gene expressions using the molecular timetable method. To select time-indicating genes that were definitely expressed periodically, we calculated the correlation value (*r*) which indicates periodicity, and amplitude value (*a*) for 18,332 genes (see Materials and Methods). The histograms showed that neither *r* nor *a* values were normally distributed (Figures 2A,B) and more than half of all genes had *r* < 0.5. About 5% of all genes exhibited clear periodic expression. In contrast, the histogram of *a* values showed that *a* values were distributed around 0.5 and spread widely around high value. About 70% of all genes had *a* values within 0–1. The heat map of 1132 periodic genes, which indicated periodicity and high amplitude through setting of cut-off values of *r* = 0.80 and *a* = 0.15, clearly showed a diagonal striped pattern, indicating that we could correctly select the periodic genes (Figure 2C). We showed the expression profiles of 143 time-indicating genes through setting the cut-off values of *r* = 0.915 and *a* = 0.15 for each time point on the 1st and 2nd days (Figure 2D). The vertical axis represents the normalized expression level and the horizontal axis represents the molecular peak time. Thus, Figure 2D shows the overall behavior of 143 time-indicating genes at each sampling time. Genes related to photosynthesis, translocation of sugar and photoreceptor were pooled in 143 time-indicating genes and the timing of their expression was consistent with their physiological function (Table S2). As time proceeded, the peak position shifted at regular intervals. Furthermore, the expression profiles on the 1st and 2nd days were similar in most respects. This indicates that these 143 genes showed stable periodic expression at daily intervals under fluctuating field conditions. We plotted normalized expression levels for the 1st and 2nd days to verify stability (Figure 2E). All genes were plotted in a linear fashion despite the 2nd day being rainy. The determination coefficient (*R*^2^) between them was high (*R*^2^ ≈ 0.7; Figure 3B). Thus, this supports the claim that time-indicating genes were stably expressed under fluctuating field conditions.

### Validation of the internal time estimation

We estimated the internal time from the expression profiles of 143 time-indicating genes for each time and calculated the difference between time and estimated internal time (Figure 3A). Internal time showed much the same time as the sampling time; however, there was a difference of more than 1 h between the two for some sampling points. As an additional indication of stability, we showed the transition of R^2^ between normalized expression levels of the 1st and 2nd days (Figure 3B). There was a high correlation indicated by high *R*^2^ values (>0.65) at all times.

### Diurnal gene expression profiles of stress-responsive genes

**Figure 4:**
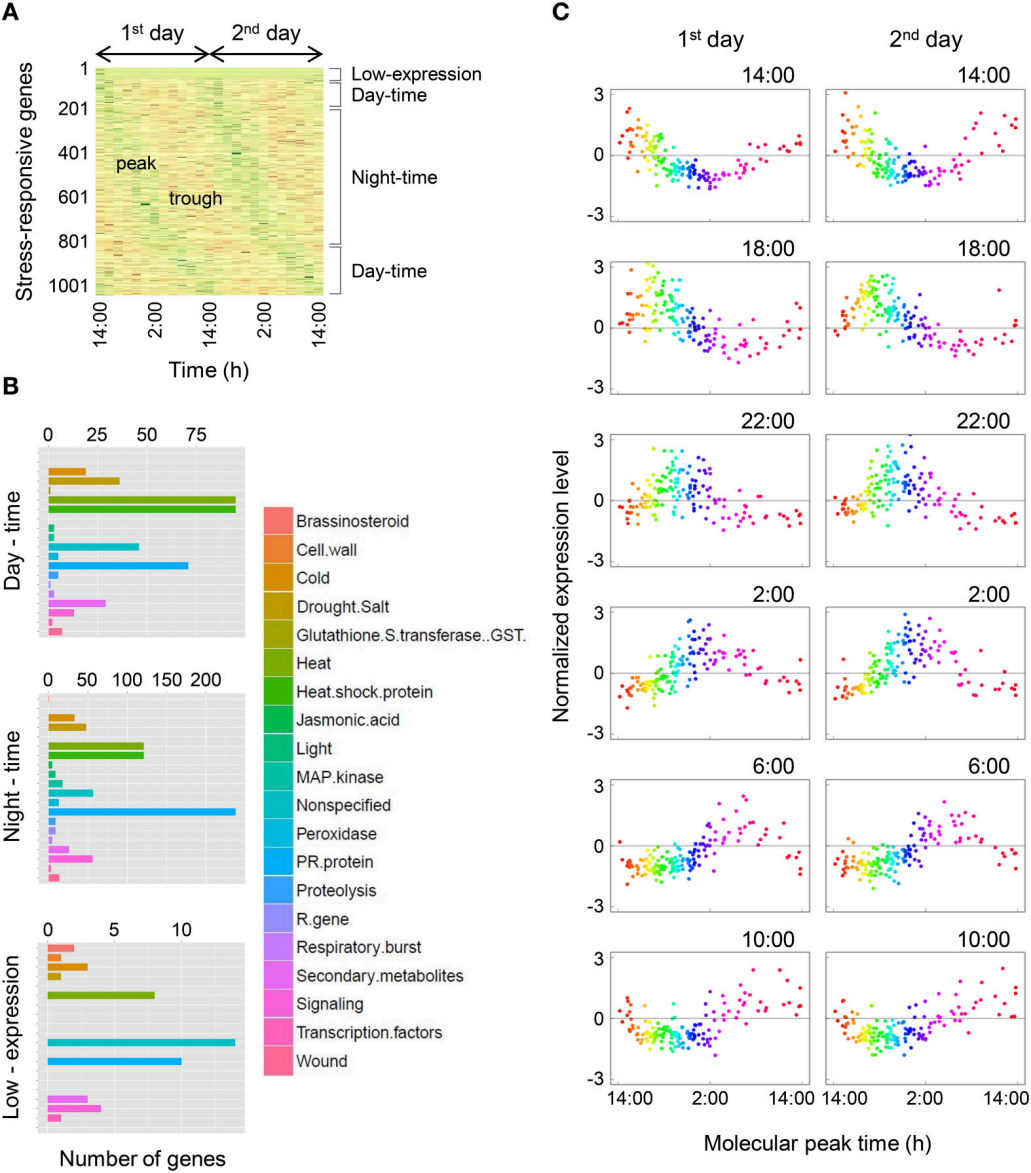
**Diurnal gene expression profiles of stress-responsive genes. (A)** Oscillation behavior of stress-responsive genes. Green areas indicate the peak and red areas indicate the trough. **(B)** Categorization of stress-responsive genes using MapMan. Day-time was 7:00–17:00 (10 h) and night-time was 17:00–7:00 (14 h). **(C)** Expression profiles of 150 time-indicating genes for the 1st and 2nd days in stress-responsive genes. The time at the top right of the plot area indicates the sampling time. The range from orange to purple represents night-time and other colored areas represent day-time.

We focused on the stress-responsive genes, which are important to increase sweetness in tomato fruit and to defend against disease. Furthermore, stress-responsive genes are possible markers of the internal factors because these genes show sensitivity to external stimuli and affect several physiological events (Wang et al., 2003; Chinnusamy et al., 2005; Umezawa et al., 2006; Cattivelli et al., 2008). We extracted 1048 stress-responsive genes from all genes using the MapMan and analyzed them by the molecular timetable method. The heat map of the 1048 stress-responsive genes indicated that they were expressed periodically under fluctuating field conditions without the addition of external stress such as heat or drought during the experiment (Figure 4A). Then we separated the stress-responsive genes into three types—day-time (434 genes), night-time (786 genes), and low-expression (47 genes)—according to the molecular peak times, and categorized them using the MapMan (Figure 4B). Day-time was 7:00–17:00 (10 h) and night-time was 17:00–7:00 (14 h). More than half of the stress-responsive genes had expression peaks at night in tomato leaves. Genes related to cold, heat, and drought stress had expression peaks equally during day and night. Then we showed the expression profiles of 150 time-indicating genes, which indicated periodicity and high amplitude, through setting the cut-off values of *r* = 0.635 and *a* = 0.15 in the stress-responsive genes (Figure 4C). These expression profiles were clearly noisy compared with expression profiles of the 143 most-periodic time-indicating genes (Figure 2D); however, there was certainly periodicity without stress being applied.

## Discussion

At least 1000 genes, included some clock-related genes (Presentation S1), indicated periodic expression clearly in tomato leaves under agricultural situations in winter. In addition, 143 time-indicating genes indicated stable periodic expression even though the alterations in the environment had a possibility to affect such aspects of plant physiology as photosynthesis and sucrose metabolism (Feugier and Satake, 2013; McCormick and Kruger, 2015), and so the expression of relevant genes would likely be influenced by external stimuli (Nagano et al., 2012). This mediation system seemed to be generated by the internal oscillator of the circadian clock. Although 143 time-indicating genes indicated stable periodic expression, some estimated internal time showed little difference between the sampling times. It suggested that the phase of periodic genes, or of genes downstream, had their circadian clock precisely regulated to adjust to a non-constant environment and so kept their periodicity (Presentation S1). Thus, the periodicity of these genes was sufficiently stable, although there were some differences in expression level. Circadian stability was reported in rice leaves under fluctuating field conditions (Matsuzaki et al., 2015). In the case of rice, the difference between sampling times and estimated internal times was ≤ 22 min and gene expression was not affected by external factors. These differences between tomato and rice may be due to species differences. Circadian clocks exist in almost all living organisms and characteristics of plant circadian rhythms have been studied in Arabidopsis (Nakamichi et al., 2004); however, characteristics of circadian rhythm in *Lactuca sativa* differed from Arabidopsis (Higashi et al., 2014). Furthermore, tomato leaves may have an unusual circadian clock system because they develop a detrimental leaf injury when grown under constant light (Cushman and Tibbitts, 1998; Velez-Ramirez et al., 2011). One characteristic of circadian clocks is that they generate circadian rhythm under continuous conditions. This suggest that an unusual circadian clock system in tomato affected expression of the clock genes or downstream genes and resulted in the difference of estimated internal time. Recently, the inclusive CCA1 target genes in Arabidopsis were reported (Nagel et al., 2015). Then we located orthologs of the time-indicating genes using the KEGG database and identified how many orthologs of the CCA1 target genes were present in the time-indicating gene pool of tomato. We identified 23 CCA1 target genes among the time-indicating genes, accounting for about 16% of 143 time-indicating genes. This suggests that a number of time-indicating genes were expressed periodically depending on the day–night environment or influence of other clock genes, at least in this experiment. This low proportion of CCA1 target genes to time-indicating genes in tomato might affect the difference in estimated internal time. On the other hand, to detect the accurate internal time is important because plant circadian rhythms can be controlled by external stimuli (Barak et al., 2000; Covington et al., 2008; Fukuda et al., 2013). Thus, the timing of environmental control such as light supplement and temperature control affect the circadian rhythms and therefore the dimension of physiological events. It has possibilities to enhance growth and floral induction.

Stress-responsive genes were also indicated for periodic expression. Thus the stress-responsive genes were normally expressed periodically as regulated by light–dark cycle as the external factor or the circadian clock as the internal factor, and they could be specifically expressed when some stresses were experienced by the plant. Similar events were also reported for soybean, barley, and Arabidopsis (Covington et al., 2008; Habte et al., 2014; Marcolino-Gomes et al., 2014). It suggests that circadian regulation of stress-responsive genes expression may be widely-conserved systems and it provides some advantage that plant survive under fluctuating field conditions (Goodspeed et al., 2012, 2013; Grundy et al., 2015). Furthermore, this result suggests that stress-responsive genes may be competent markers for agriculture because they are simple to apply although normally expressed periodically and especially transiently.

In conclusion, we demonstrated that many genes were expressed periodically and that gene expression was stable in a sunlight-type plant factory. Furthermore, internal time could be estimated from time-course gene expression data in tomato leaves through molecular timetable method. The results also showed that stress-responsive genes were expressed periodically under non-stressed conditions. This study suggests that circadian clock mediate the optimization for fluctuating environments and environmental control tailored to internal time may enhance resistibility to stress and floral induction, eventually the yieldability.

## Author contributions

HF and TH designed the experiments. YT did the MapMan analysis and constructed the heat map. KT supported the sampling in a sunlight-type plant factory. AN and MH prepared a RNA-Seq library. TH performed RNA-Seq data analysis and the molecular timetable method. TH and HF wrote the manuscript. All authors discussed the results and implications and commented on the manuscript.

### Conflict of interest statement

The authors declare that the research was conducted in the absence of any commercial or financial relationships that could be construed as a potential conflict of interest.
