# Supplementary material for: Detection of Diurnal Variation of Tomato Transcriptome through the Molecular Timetable Method in a Sunlight-Type Plant Factory
Source: Front Plant Sci. 2016 Feb 8;7:87. doi: 10.3389/fpls.2016.00087 (PMC4744910; doi:10.3389/fpls.2016.00087)
Supplement: Supplementary file 4 [file Presentation1.PPTX]

## Slide 1
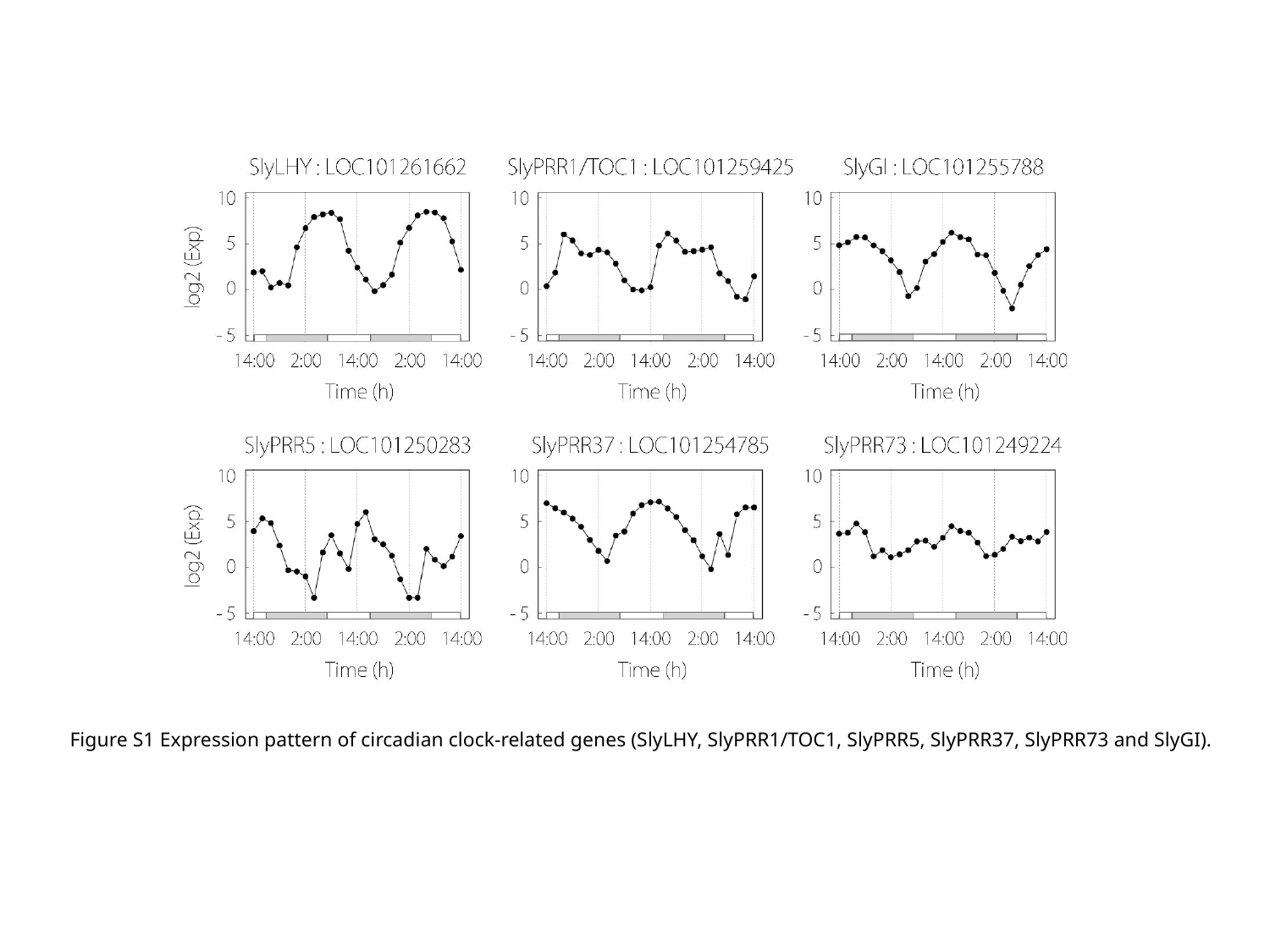

Figure S1 Expression pattern of circadian clock-related genes (SlyLHY, SlyPRR1/TOC1, SlyPRR5, SlyPRR37, SlyPRR73 and SlyGI).
